# Shape Ontogeny of the Distal Femur in the Hominidae with Implications for the Evolution of Bipedality

**DOI:** 10.1371/journal.pone.0148371

**Published:** 2016-02-17

**Authors:** Melissa Tallman

**Affiliations:** Department of Biomedical Sciences, Grand Valley State University, Allendale, MI, United States of America; University of Florence, ITALY

## Abstract

Heterochrony has been invoked to explain differences in the morphology of modern humans as compared to other great apes. The distal femur is one area where heterochrony has been hypothesized to explain morphological differentiation among Plio-Pleistocene hominins. This hypothesis is evaluated here using geometric morphometric data to describe the ontogenetic shape trajectories of extant hominine distal femora and place Plio-Pleistocene hominins within that context. Results of multivariate statistical analyses showed that in both *Homo* and *Gorilla*, the shape of the distal femur changes significantly over the course of development, whereas that of *Pan* changes very little. Development of the distal femur of *Homo* is characterized by an elongation of the condyles, and a greater degree of enlargement of the medial condyle relative to the lateral condyle, whereas *Gorilla* are characterized by a greater degree of enlargement of the lateral condyle, relative to the medial. Early *Homo* and *Australopithecus africanus* fossils fell on the modern human ontogenetic shape trajectory and were most similar to either adult or adolescent modern humans while specimens of *Australopithecus afarensis* were more similar to *Gorilla/Pan*. These results indicate that shape differences among the distal femora of Plio-Pleistocene hominins and humans cannot be accounted for by heterochrony alone; heterochrony could explain a transition from the distal femoral shape of early *Homo/A*. *africanus* to modern *Homo*, but not a transition from *A*. *afarensis* to *Homo*. That change could be the result of genetic or epigenetic factors.

## Introduction

Heterochrony has often been invoked to explain differences in the morphology between early hominins and modern humans. Heterochrony is change in the rate or timing of growth and development from a parent species (or individual) to a descendent species (or individual) [1], [2]. Heterochrony can be divided into two categories: paedomorphosis, where the descendent species retains characteristics of the juveniles in the parent species; and peramorphosis, where the descendent species appears to extend the development of the parent species. There are three classes of changes to developmentally accomplish paedo- or peramorphosis: (1) progenesis or hypermorphosis, where maturation with respect to the trait is reached early in the former and continues late in the latter; (2) neoteny or acceleration, where development occurs more slowly in the former and more rapidly in the latter; and (3) pre-displacement or postdisplacement, where development begins later in the former and earlier in the latter [2], [3]. One of the first examples of heterochrony being invoked in human evolution is from the discussion of human cranial shape. Modern humans were sometimes considered neotenous for cranial shape in comparison to fossil hominins and other hominoids [1], [4], although that explanation has since been found to be an oversimplification of the developmental processes that shape the human cranium [5], [6]. Developmental changes in the dentition between humans and great apes have also been explained by time hypermorphosis in *Homo* [7].

The distal femur is a biomechanically important joint in locomotion [8], and the shape of the distal femur is a clear marker for the acquisition of bipedal posture [8], [9]. This–in addition to its representation in multiple fossil hominin taxa–makes the distal femur an important region for study in Plio-Pleistocene hominins. The shape of the distal femur in fossil hominins has been analyzed utilizing discrete characters, linear measurements [10], [11], [12], [13], two dimensional geometric morphometrics [14], and three-dimensional geometric morphometrics [15],[16], and conclusions from these analyses have largely been used to make inferences about the taxonomy of fossil hominins and/or their biomechanical capability at the knee joint. Fewer studies have investigated the potential underlying causes for shape variability among fossil hominin distal femora.

Lovejoy et al. [8] argued that the distinctive shape of the human distal femur could be the result of modifications to genes that regulate chondral growth fields. These authors suggested that only a small genetic modification to these growth fields, in combination with the biomechanical demands of upright walking, could produce the entire suite of traits that differentiates the human knee from those of other extant apes. Conversely, Tardieu [12], [13] hypothesized that the cause of distal femoral shape variation among Plio-Pleistocene hominins was due to heterochrony–specifically peramorphosis–from *Australopithecus* to early *Homo* and from early *Homo* to modern humans. She suggested that there was a lengthening of the adolescent growth period in *Homo*. Using radiographs, she compared the shape of the distal articular surface and the profile of the lateral condyle of fossil hominins to a human developmental series. She found A.L. (Afar Locality) 129-1a to look like a human child aged 10 years, whereas KNM-ER (Kenya National Museum–East Rudolf) 1472 was most similar to a late adolescent aged 16 years and KNM-ER 1481 was most like a 17 year old. However, it should also be noted that Tardieu [17] concluded that the bony morphology of children are not indicative of their functional morphology; the true functional morphology of a child’s knee is reflected in the cartilaginous structure, which is similar to the adult form. Finally, Tardieu [12], [13] also showed that an adult chimpanzee was similar in shape to a human child. These data agree with findings by Berge [18] who demonstrated that the shape of the human ilium could also be the result of peramorphosis.

This study investigates the idea that the shape of the modern human distal femur is the result of heterochrony by using three-dimensional geometric morphometric techniques to quantify the shape changes in the distal femur through ontogeny. Thus, this study has two major goals:

1. Describe and quantify the ontogenetic shape changes that occur in the distal femora of *Pan*, *Gorilla* and *Homo*.
2. Place Plio-Pleistocene hominins within the context of this variation to evaluate whether their distal femoral shapes can be accommodated within the ontogenetic shape trajectories of any of these taxa.

If fossil hominins can be accommodated within the modern human ontogenetic shape trajectory, then this would fail to reject the hypothesis that the modern human distal femoral shape is due to heterochrony. If fossil hominins are best accommodated by the *Pan* or *Gorilla* shape trajectory, and the *Pan* or *Gorilla* shape trajectories are statistically similar to the modern human trajectories, then this would also fail to reject this hypothesis. If the ontogenetic shape trajectories of the great apes and humans are statistically different, and hominins do not fit the human trajectory, then the hypothesis is rejected.

## Materials and Methods

**Fig 1:**
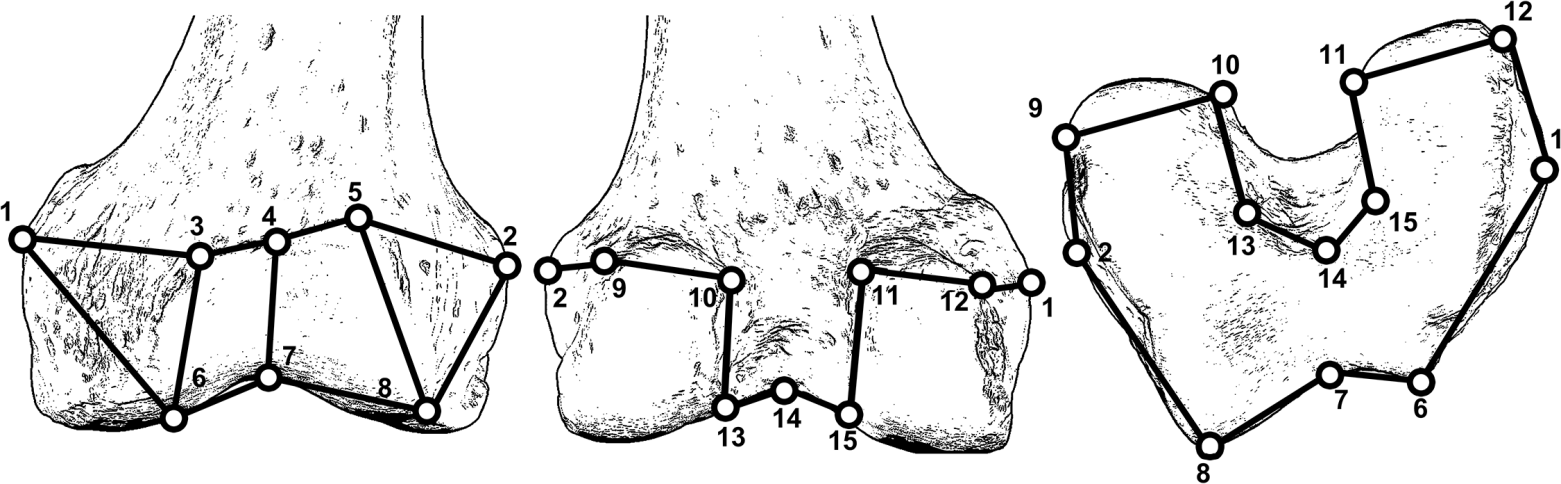
Diagram illustrating the landmarks used on the distal femur in anterior (left) posterior (middle) and distal (right) views. Thick black lines are wireframes for visualization purposes only. A human femur is used for illustrative purposes.

Three-dimensional geometric (3D-GM) data were collected to characterize the morphology of the distal femur. This morphometric approach allows for the retention of shape information for statistical analyses by using data in the form of *x*,*y*,*z* coordinates (landmarks). This allows for the visualization of changes among the original specimens in analyses [19]. Fifteen landmarks (Fig 1 and Table 1) were collected using a Microscribe 3DX digitizer on the distal femora of original fossils and an ontogenetic series of *Homo sapiens* (housed at the Musée de L’Homme, Paris, France), *Pan troglodytes troglodytes* and *Gorilla gorilla gorilla* (both housed at the Powell-Cotton Museum, Birchington, UK). The distal femur is an ideal region to use for these kinds of analyses as it is one of the few areas in the postcranial skeleton that develops a secondary center of ossification prior to birth [12], [20] and thus is present in skeletal collections of young individuals. If the distal epiphysis was completely unfused, it was attached to the diaphysis based on the fit of the congruent surfaces by a thin piece of clay such that it would not move during data collection. The age and sex of the *Homo* individuals were recorded from the museum catalogue. Where data on sex were not available, sex was phenetically assessed by pelvic morphology; specifically, the width of the sciatic notch, the width of the subpubic angle, and the shape of the anteriormost aspect of the pubis were used. In all cases where sex could not be confidently ascertained, or if the pelvis was missing, it was scored as unknown. For *Pan* and *Gorilla*, epiphyseal closure and dental eruption were used as a proxy for age and scored on a 1 to 4 scale (Table 2) (*Pan* tooth eruption data from [21]; *Gorilla* tooth eruption data from [22]) while sex was recorded from the museum catalogue. All *Pan* and *Gorilla* were wild shot and displayed no obvious pathologies. Sampling of individuals was restricted to a single subspecies for *Pan* and *Gorilla* to eliminate any possible variability in ontogenetic shape development at the subspecific or specific level. Sampling of adults was restricted so as not to statistically bias any results towards the adult morphology. Sampling of fossil individuals was restricted to those that had complete distal femora (Table 3).

**Table 1:** Description of the landmarks taken on the distal femur.

Number	Type	Description
1	III	most medial point on medial epicondyle
2	III	most lateral point on lateral epicondyle
3	III	most proximomedial point on the proximal border of the patellar articular surface on the anterior aspect of the distal femur.
4	III	midpoint between the most proximomedial and proximolateral point on the proximal border of the patellar articular surface on the anterior aspect of the distal femur.
5	III	most proximolateral point on the proximal border of the patellar articular surface on the anterior aspect of the distal femur.
6	III	most distomedial point on the distal border of the patellar articular surface on the anterior aspect of the distal femur.
7	III	midpoint between the most distomedial and distolateral point on the distal border of the patellar articular surface on the anterior aspect of the distal femur.
8	III	most distolateral point on the proximal border of the patellar articular surface on the anterior aspect of the distal femur.
9	III	most posteriomedial point on medial condyle
10	III	Most posteriolateral point on medial condyle
11	III	most posteriomedial point on the lateral condyle
12	III	most posteriolateral point on the lateral condyle
13	III	most medial point in the middle of the intracondylar notch
14	III	middle point in the middle of the intracondylar notch
15	III	most lateral point in the middle of the intracondyle notch
16	III	most medial corner on the anterior edge of the intracondylar notch
17	III	middle point on the anterior edge of the intracondylar notch
18	III	lateral corner on the anterior edge of the intracondylar notch.

**Table 2:** Description of the extant ontogenetic sample.

Group/Taxon	Age Class	Definition	n
Juvenile *Pan*	1	No epiphyses fused; M1 erupting	15
Early Adolescent *Pan*	2	Some epiphyses partially fused; M1 erupted, M2 erupting	7
Late Adolescent *Pan*	3	Some epiphyses fully fused; M2 erupted through M3 erupting	19
Adult *Pan*	4	All epiphyses fused, M3 erupted	4
Juvenile *Gorilla*	1	No epiphyses fused; M1 erupting	9
Early Adolescent *Gorilla*	2	Some epiphyses partially fused; M1 erupted, M2 erupting	15
Late Adolescent *Gorilla*	3	Some epiphyses fully fused; M2 erupted through M3 erupting	5
Adult *Gorilla*	4	All epiphyses fused, M3 erupted	5
Juvenile *Homo*	1	Ages 2–7	12
Early Adolescent *Homo*	2	Ages 8–14	6
Late Adolescent *Homo*	3	Ages 14–20	5
Adult *Homo*	4	Over 25	4

**Table 3:** Description of the fossil sample.

Accession Number	Taxon	Repository
A.L. (Afar Locality)129-1a ^1^	*Australopithecus afarensis*	National Museum of Ethiopia
A.L. 333–4 ^1^	*Australopithecus afarensis*	National Museum of Ethiopia
KNM-ER (Kenya National Museum–East Rudolf)1472 ^2^^,^^3^^,^^4^	*Homo sp*.	Kenya National Museum
KNM-ER 1481 ^2^^,^^3^^,^^4^	*Homo sp*.	Kenya National Museum
KNM-ER 1592 ^5^	Hominidae *sp*. *indet*.	Kenya National Museum
KNM-ER 3951^5^	Hominidae *sp*. *indet*.	Kenya National Museum
KNM-WT (Kenya National Museum–West Turkana)15000 ^6^	*Homo erectus*	Kenya National Museum
Omo 1^7^	*Homo sapiens*	National Museum of Ethiopia
Sts (Sterkfontein South) 34 ^5^^,^^8^	*Australopithecus africanus*	University of Witswaterstrand

^1^[69]

^2^[11]

^3^[70]

^4^[71]

^5^[72]

^6^[73]

^7^[74]

^8^[75]

Two precision tests were completed before data collection for this study. In the first test, ten replicate landmark sets were collected on adult, white human males housed in the Department of Anthropology at the American Museum of Natural History. Data on these replicates were collected over the course of a week, and the bone was unmounted and remounted between each replicate. Data on these specimens were then subjected to a generalized Procrustes analysis (GPA) which rotates, translates, and scales landmark configurations by minimizing the sum of squares distance between them [19]. The Procrustes distance between each replicate and the mean landmark configuration was calculated. Procrustes distance is the square root of the sum of squared differences between all landmarks in a pair of individuals [23]. Subsequently, data were collected on ten different adult white males from the same collection. These data were then subjected to a GPA, and Procrustes distances from each individual to the consensus landmark configuration were calculated. Results from t-tests indicated that the mean Procrustes distance among replicates of the same specimen were significantly smaller than the distances between ten different specimens of the same sex from the same population (Table 4).

**Table 4:** Average pairwise Procrustes distances (*d)* for the entire landmark configuration between 10 repeated trials on the same specimen (rep), and between 10 different individuals from the same population (x).

Rep.	*d*	x	*d*
1	0.0105839	1	0.027592
2	0.0097056	2	0.020099
3	0.0080857	3	0.02265
4	0.0111851	4	0.022731
5	0.0087269	5	0.019499
6	0.0078037	6	0.026027
7	0.0127367	7	0.029197
8	0.0102955	8	0.021333
9	0.0100383	9	0.027688
10	0.0093378	10	0.022137
**AVERAGE**	**0.00985**		**0.0239**
***p* = < 0.0001**			

In order to assess the variability at each individual landmark, the Procrustes distances from each individual landmark to the consensus landmark were calculated for both the ten replicates and the ten different individuals from the dataset above. The mean and variance for the distribution of Procrustes distances in both samples were calculated; these data are recorded in Table 5. T-tests indicated that the average Procrustes distances at each landmark were all significantly smaller for the replicates than for ten different individuals.

**Table 5:** Average pairwise Procrustes distances, and average variance, for each landmark between 10 repeated trials on the same specimen (rep), and between 10 different individals from the same population (x).

	average		variance	
	rep	x	rep	x
1	0.0020	0.0039	0.0000010	0.0000034
2	0.0028	0.0046	0.0000010	0.0000024
3	0.0007	0.0028	0.0000001	0.0000026
4	0.0012	0.0024	0.0000003	0.0000004
5	0.0017	0.0041	0.0000006	0.0000034
6	0.0017	0.0030	0.0000004	0.0000023
7	0.0011	0.0023	0.0000004	0.0000018
8	0.0025	0.0044	0.0000026	0.0000036
9	0.0007	0.0049	0.0000001	0.0000061
10	0.0008	0.0027	0.0000002	0.0000015
11	0.0007	0.0025	0.0000000	0.0000018
12	0.0010	0.0033	0.0000004	0.0000032
13	0.0008	0.0028	0.0000002	0.0000026
14	0.0009	0.0021	0.0000002	0.0000003
15	0.0008	0.0025	0.0000002	0.0000020
	p = 0.0001		p = 0.0001	

The landmark coordinates were subjected to a GPA. The GPA included all specimens at all ages in order to get a common fit among the different species and age classes [24]. A principal components analysis (PCA) was conducted on the full sample in Procrustes shape space. A PCA of the full sample allows for the exploration of variation in both total shape and ontogenetic shape in a common space. In a mixed-species ontogenetic sample, the first principal component will be variation that comprises both the common aspects of the different allometric trajectories and some species specific differences [25].

In order to determine how much shape variation is correlated with differences in size in the extant taxa, a multivariate regression of shape coordinates on ln-centroid size was computed for each taxon in MorphoJ [26]. The percent of the shape variability explained by centroid size for each taxon was recorded and shape changes correlated with centroid size were visualized. Differences in the degree of ontogenetic change for each taxon were examined by looking at the correlation between shape change and size change from the average juvenile individual. Shape change was calculated as the ln-Procrustes distance between the average juvenile and each individual, and size change was calculated as the difference in ln-centroid size from each individual to the average juvenile. The slope of the line from a univariate regression of change in ln-Procrustes distance and change in ln-centroid size is a representation of the degree of developmental shape change that occurs in each taxon. If a taxon does not change in shape as it grows, the slope of the line will approach zero [27]. A similar procedure was used by Kim et al. [28] to examine patterns of development in two species of trilobites and by Zelditch et al. [29] in a study of piranhas. This analysis tests whether the degree of shape change that is correlated with growth is the same across all three taxa. A separate analysis was performed to assess whether there were differences in rates of shape change in males and females of each taxon, but as the slopes and intercepts of the lines were similar in all cases, these results are not presented here.

If two ontogenetic trajectories for the entire shape of the distal femur differ only due to heterochrony, they should overlap in size-shape space, and only differ in terms of length and size differences associated with the length of the developmental trajectory. As such, a PCA of the Procrustes-aligned coordinates alone is not sufficient to make inferences about heterochrony as it does not sufficiently describe the totality of size/shape space [25], [30]. In order to determine if the ontogenetic trajectories of *Pan*, *Homo*, and *Gorilla* overlap, a series of ten configurations for each taxon were generated from the multivariate regressions of the Procrustes-aligned shape coordinates on ln-centroid size. These ten configurations were generated at even intervals over the entire range of size variation in each taxon. An “ontogenetic PCA” was subsequently performed on these data, and scores for all of the individuals in the analysis–including fossils–were computed posthoc based on the covariance matrix, eigenvalues, and eigenvectors of the PCA of the hypothetical ontogenetic series. This post hoc computation of scores allows for the “projection” of the extant and fossil taxa into this space. This creates a morphospace where the variation present in the sample is distributed based on the direction of maximum variability in developmental trajectory among the sampled taxa. This method was developed and used by Mitteroecker et al. [30] in order to examine heterochrony in the cranium of *Pan troglodytes* and *Pan paniscus* using 3D geometric morphometric data. If the ontogenetic trajectories at least partially overlap in this analysis, the hypothesis of species differences due to heterochrony cannot be rejected. If differences in the shape of fossil hominins relative to each other and modern humans are caused by heterochrony, the distribution of hominins will overlap the ontogenetic trajectory of modern humans [30]. Three principal component (PC) axes were visualized in the resulting graph in order to better represent the complexity of the shape space and were rotated to a position where differences among the taxa were most obvious [25], [30].

As a final measure of shape similarity, Procrustes distances between each fossil hominin and the average individual in each taxon age class were also calculated. Individuals were classified into four different age classes–juvenile, early adolescent, late adolescent, and adult–based on tooth eruption and epiphyseal closure (Table 2). The smaller the Procrustes distance, the more similar the fossil to that group average.

## Results

**Fig 2:**
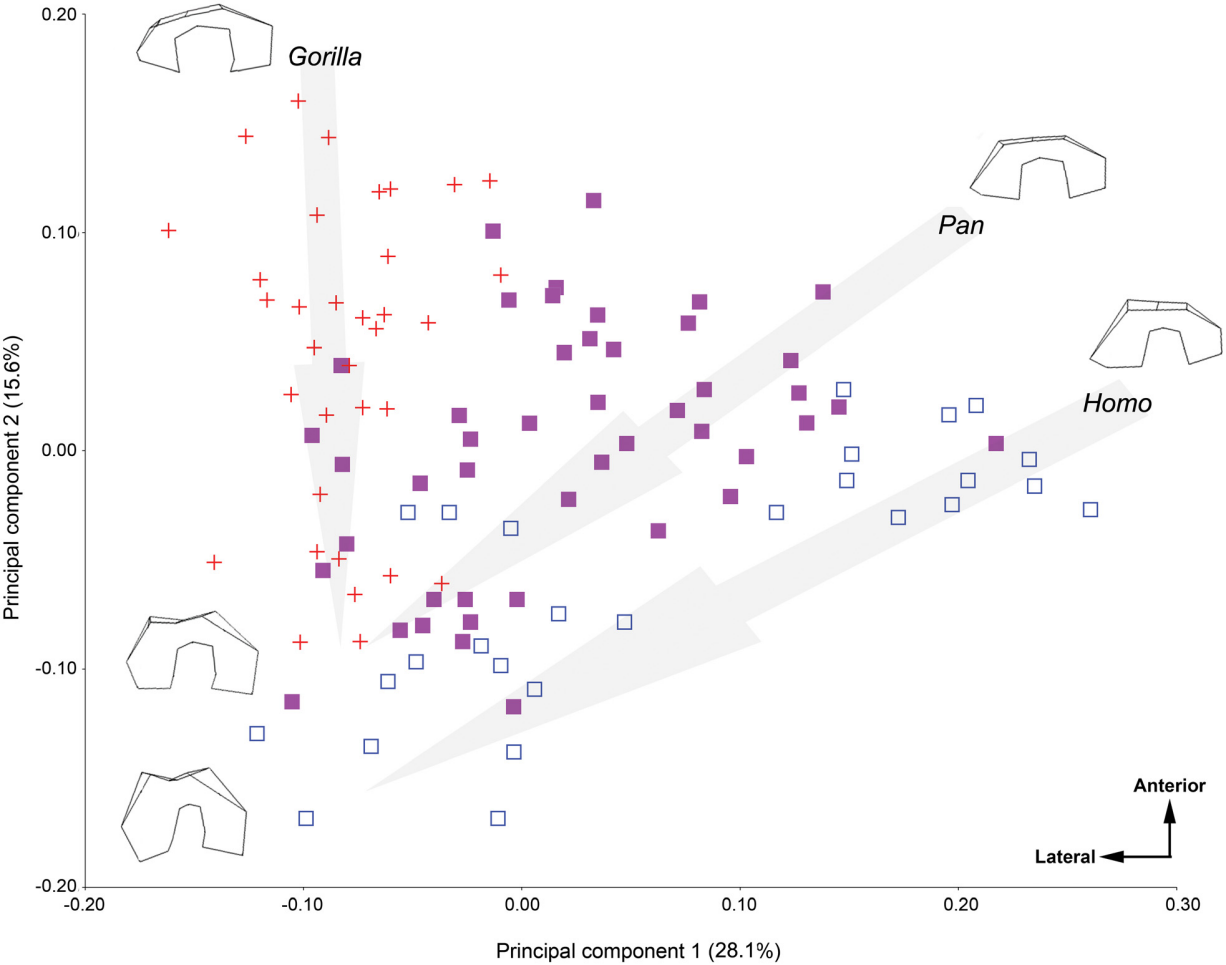
PCA in Procrustes shape space of the Procurstes-aligned data for the entire sample. Wireframes are of a left femur, illustrate the shape changes in the graphs from juvenile individuals to adult individuals and are shown in distal view. Wireframe of adult *Pan* is omitted as the adult individuals fell quite near to adult *Gorilla* in this plot. Arrows are drawn for illustrative purposes from the youngest age classes to the oldest age classes. *Gorilla* is represented by red crosses, *Pan* by purple squares, and *Homo* by blue open squares.

Fig 2 illustrates the results a PCA on the entire sample in Procrustes shape space. PC 1 is driven by the size of the medial condyle relative to the lateral condyle; young *Homo* occupy the most positive values on PC 1 and have small medial condyles in comparison to the lateral condyle whereas young *Gorilla*, adult *Homo*, adult *Pan*, and adult *Gorilla* occupy more negative values as they have larger medial condyles. PC 1 also accounts for the anteroposterior length of the distal articular surface, particularly between the deepest point in the intercondylar notch and the patellar articular surface. Young *Homo* and *Pan* have shortened distal articular surfaces, whereas adult members of all three taxa and young *Gorilla* have longer distal articular surfaces. PC 2 is driven by the keeling of the patellar articular surface and the relative size of the lateral condyle. Young *Gorilla* occupy the most positive values and have the least keeled patellar articular surfaces and the smallest lateral condyles whereas adult *Homo* occupies the most negative values on PC 2 and has the most keeled articular surface and the largest lateral condyles. Young *Homo*, *Pan*, and *Gorilla* overlap only slightly on PC 1 and 2, whereas adults of all three are more similar, although *Homo* is most distinct.

**Fig 3:**
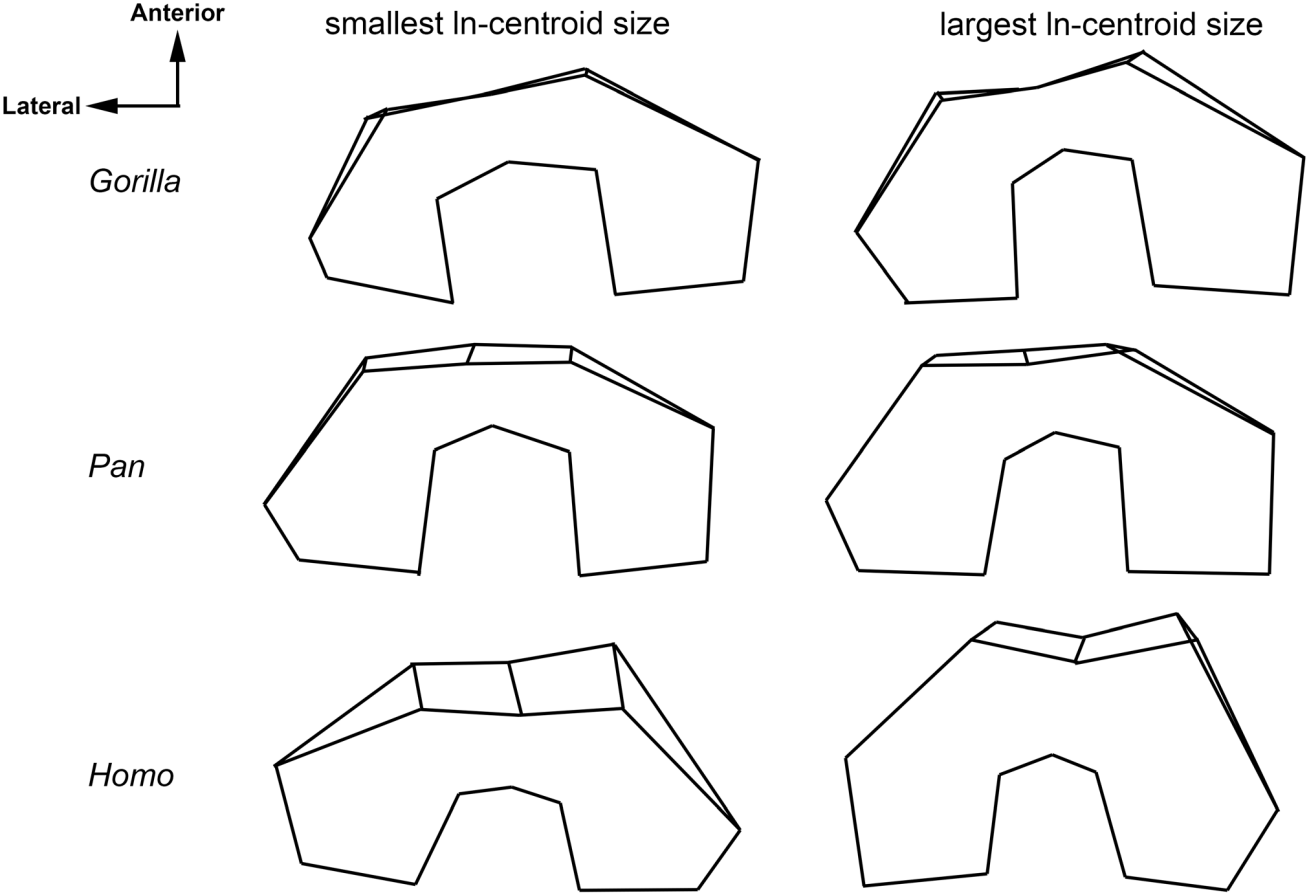
Shape changes correlated with an increase in ln-centroid size in multivariate regression analyses.

**Fig 4:**
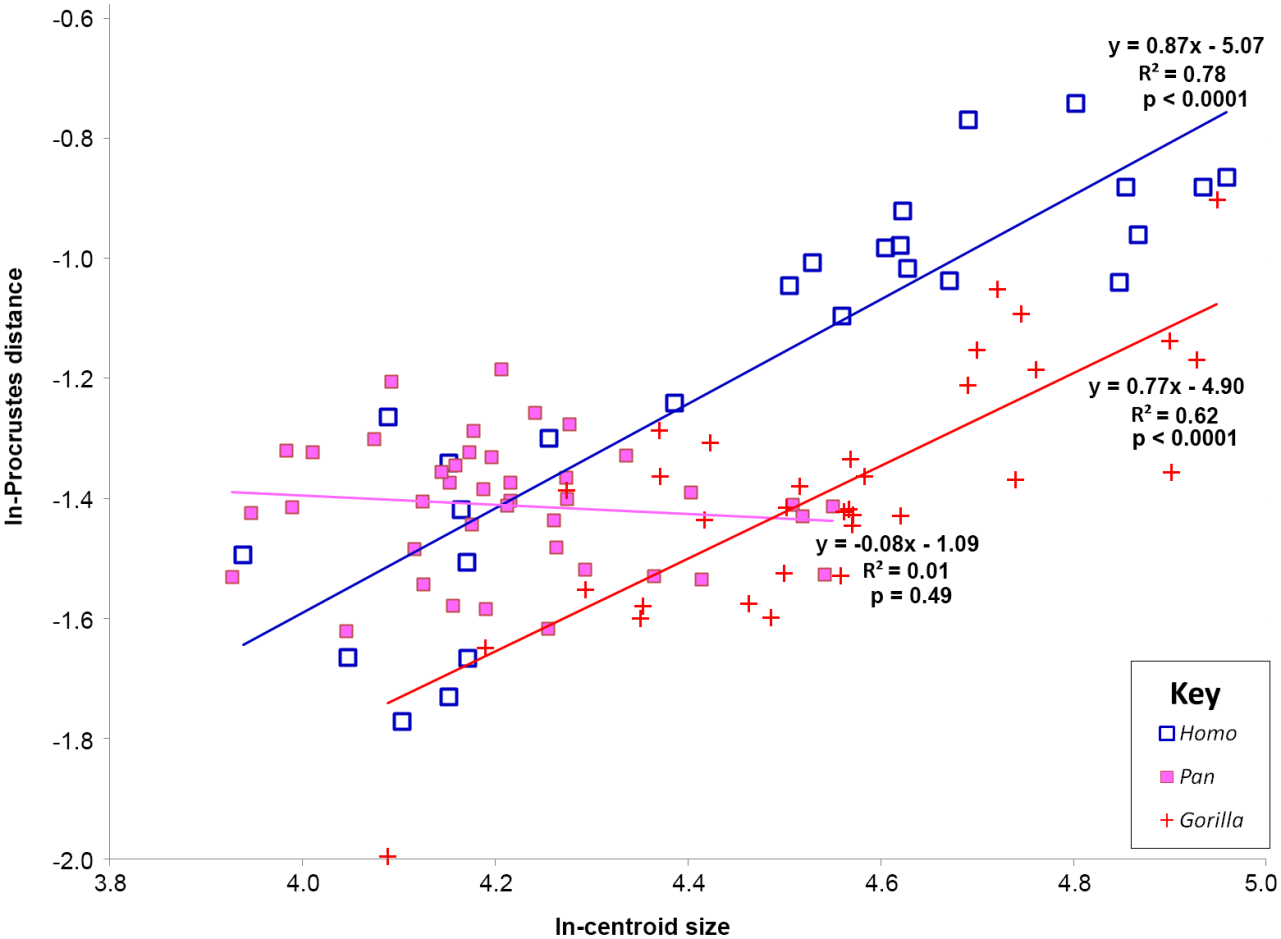
Regression of ln-Centroid size against Procrustes distance from the average youngest individual. Regressions were performed on each taxon separately and then graphed together. *Homo* is represented by blue open squares, *Pan* by pink squares, and *Gorilla* by red crosses. The thick red line represents the regression line for *Homo*, black for *Pan*, and grey for *Gorilla*. Regression equations are given in the figure.

Based on a multivariate regression of shape on ln-centroid size, *Homo* has the greatest degree of shape variation that is correlated with size at 49.0%, followed by *Gorilla* with 21.3%, and *Pan* at 8.2%. In *Homo*, an increase in size is correlated with an increase in the anteroposterior length of both the medial and lateral condyles as well as an increase in the keeling of the patellar articular surface (Fig 3). In *Gorilla*, an increase in size is associated with an increase in the anteroposterior length and proximodistal height of the medial and lateral condyles and decrease in the mediolateral width of the intercondylar notch (Fig 3). Finally, in *Pan*, an increase in size is associated with an increase in the mediolateral width of the medial and lateral condyles, an increase in height in the patellar articular surface, and a decrease in the mediolateral width of the intercondylar notch (Fig 3). Similarly, the degree of shape change in the distal femora of both *Gorilla* and *Homo* is significantly correlated with changes in size (Fig 4). *Gorilla* and *Homo* change in shape as they change in size at similar rates as evidenced by the similar slope of their regression lines. In *Pan*, there is no significant relationship between shape change and size change indicating that the adult shape of the *Pan* distal femur does not differ as much from the juvenile shape as in the other two taxa.

**Fig 5:**
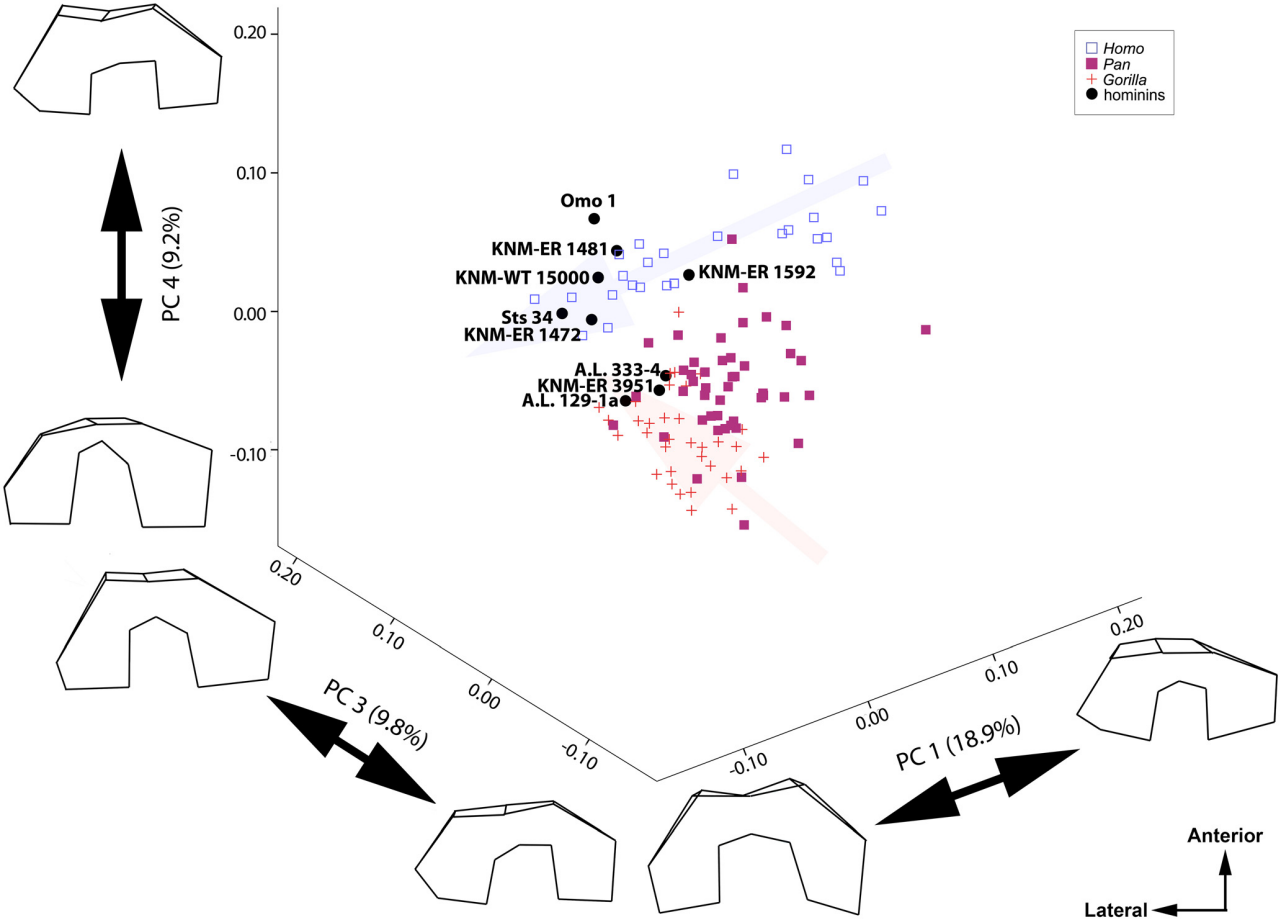
Ontogenetic PCA illustrating the position of the modern taxa and fossil individuals. Scatterplot is of components 1, 3, and 4. *Pan* is represented by pink solid squares, *Gorilla* by red crosses, and *Homo* by blue open squares. Fossils are represented by black circles and labeled in the graph. Femora are shown in distal view.

Fig 5 illustrates a PCA of the extant and fossil specimens projected into a morphospace defined by the resampled developmental trajectories of the extant groups. The shape changes associated with the three developmental trajectories are similar to those changes seen in the PCA of the Procrustes aligned coordinates (Fig 2). The shape changes along PC 1 are driven largely by the developmental trajectory of *Homo*; younger individuals at the most positive values have anteroposteriorly short femoral condyles and shorter, less keeled patellar articular surfaces. Older individuals at the most positive values have longer femoral condyles and taller, more keeled patellar articular surfaces. The shape changes along PC 3 are largely driven by the developmental trajectory of *Gorilla*. Younger individuals at more negative values have more symmetrical condyles whereas older individuals at more positive values have an enlarged medial condyle. PC 4 separates the entire distribution of *Homo* from *Pan* and *Gorilla* and is driven by the depth of the intercondylar notch and the size of the medial condyle; *Pan* and *Gorilla* have deep notches with enlarged medial condyles whereas *Homo* has an anteroposteriorly shallower intercondylar notch and smaller medial condyle. No axis of variation characterized specifically the developmental trajectory of *Pan*. Most of the fossil hominins attributed to the genus *Homo* are found along the *Homo* developmental trajectory, including KNM-ER 1592, KNM-ER 1481, KNM-ER 1472, KNM-WT 15000, and Omo 1. Sts 34 is also found along the *Homo* developmental trajectory. A.L. 333–4, A.L. 129-1a, and KNM-ER 3951 are found within the distribution of *Pan* and older *Gorilla* (Fig 5).

PC 2 in this analysis comprised 9.9% of the shape variation in this sample and was mainly associated with the width of the intercondylar notch and orientation of the patellar surface with respect to the tibial articular surface. Individuals with narrower intercondylar notches and more acute angles between the two articular surfaces (older *Homo* and some *Pan*) had more negative values and those with wider intercondylar notches and less acute angles between the two articular surfaces (younger *Homo* and some *Pan*) had more positive values. The overall patterning of individuals along PC 2 was similar to that of PC 1 and is thus not shown.

Table 6 lists the Procrustes distance of each fossil to the mean of each age class of each extant taxon. Most of the fossil hominins that have been classified in the genus *Homo–*KNM-WT 15000, KNM-ER 1472, KNM-ER 1481, KNM-ER 1592, and Omo 1—are most similar in shape to adult or adolescent modern humans. Sts 34 is also most similar to modern humans. A.L. 333–4 and KNM ER-3951 are most similar to adult *Gorilla*. A.L. 129-1a was equally similar to adult *Gorilla* and adult *Pan*.

**Table 6:** Procrustes distances between each fossil individual and each mean age class for each taxon.

	*Gorilla* age class 1	*Gorilla* age class 2	*Gorilla* age class 3	Adult *Gorilla*	*Homo* age class 1	*Homo* age class 2	*Homo* age class 3	Adult *Homo*	*Pan* age class 1	*Pan* age class 2	*Pan* age class 3	Adult *Pan*
A.L. 129-1a	0.247	0.187	0.151	**0.149**	0.330	0.175	0.165	0.158	0.240	0.214	0.182	0.177
A.L. 333–4	0.260	0.199	0.145	0.136	0.295	0.181	0.173	0.191	0.228	0.193	0.171	**0.130**
KNM-ER 1472	0.284	0.232	0.188	0.164	0.294	0.169	**0.141**	0.146	0.235	0.208	0.196	0.159
KNM-ER 1481	0.296	0.254	0.224	0.207	0.285	0.148	**0.116**	0.119	0.231	0.223	0.206	0.198
KNM-ER 1592	0.277	0.249	0.223	0.187	0.304	0.181	0.184	**0.179**	0.269	0.249	0.224	0.222
KNM-ER 3951	0.315	0.282	0.244	**0.207**	0.363	0.269	0.245	0.236	0.318	0.297	0.256	0.224
Omo 1	0.436	0.391	0.357	0.340	0.436	0.338	0.286	**0.278**	0.395	0.378	0.360	0.333
Sts 34	0.312	0.273	0.226	0.213	0.376	0.231	0.203	**0.187**	0.309	0.278	0.258	0.221
KNM-WT 15000	0.328	0.288	0.274	0.253	0.289	0.187	**0.181**	0.193	0.253	0.256	0.239	0.250

## Discussion

### Variability in distal femoral shape ontogeny in the great ape-human clade

Differences in the ontogenetic trajectories of the full distal femur of *Gorilla*, *Pan*, and *Homo* cannot be explained by heterochrony alone, as evidenced by the results presented in Fig 5. Although the shape of the distal femur changes significantly in both *Gorilla* and *Homo* as they age (Fig 3 and Table 3), their ontogenetic trajectories differ. In the youngest *Gorilla* and *Homo*, the medial and lateral condyles are of extremely unequal sizes; in youngest *Gorilla*, the medial condyle is much larger than the lateral condyle whereas in the youngest *Homo*, the lateral condyle is much larger than the medial. However, while condyle size inequality persists into the adult forms, they appear less unequal than in the juvenile forms (Figs 2 and 5); thus *Gorilla* development requires greater enlargement of the lateral condyle relative to the medial condyle and *Homo* development requires greater enlargement of the medial condyle relative to the lateral condyle as they age.

In *Gorilla*, adult shape could be related to the support of large body weights during locomotion [31]. Ruff [32] found that, among extant great apes, *Gorilla* had the most enlarged medial condyle as compared to lateral condyle and the most varus position of the knee. The enlargement of the medial condyle may allow for the transmission of higher loads and may assist in distributing the high compressive forces generated as a result of having a varus knee [31]. Ruff [32] suggested that the large size of *Gorilla* could have driven selection towards a knee built for weight support at the expense of speed. While chimpanzees and gorillas are often both considered under the umbrella of “knuckle-walking”, their positional behaviors are significantly different [33]. Gorillas engage in a variety of positional behaviors, but spend more time engaged in terrestrial quadrupedalism and are less acrobatic than chimpanzees both in the wild [33], [34], [35] and in captivity [36]. Besides behavioral differences between the two taxa, there are several other lines of evidence that their postcranial ontogenetic trajectories are different. For instance, their hand proportions are significantly different throughout ontogeny [37] as is the way that knuckle-walking develops behaviorally [38] and morphologically [39]. Additionally, the pattern and timing of the female growth spurt is absolutely different in the two taxa [5].

In humans, adult shape of the distal femur is likely related to the mechanical requirements of bipedality. Unlike great apes, the lateral and medial condyles of humans are of similar size because they are equally important in transmitting downward forces and resisting reaction forces during bipedal locomotion. These condyles are more elliptical (particularly the lateral condyle) and more symmetrical in order to provide a larger surface for articulation with the tibia and to reduce stress on the joint during heel-strike and toe-off during walking [9], [40]. The deep, asymmetrical patellar groove in *Homo* could function to prevent the patella from being laterally dislocated during flexion of a valgus knee [8], [10], [40], [41]. These are specializations not seen in any other mammal [40].

In *Pan*, there is little change in shape during ontogeny, and changes in shape are not well correlated with changes in size (Fig 3 and Table 3). This could be because chimpanzees are under different biomechanical constraints than *Gorilla* and *Homo* due their relatively more varied locomotor repertoire. Chimpanzees retain a variable locomotor repertoire throughout ontogeny, engaging in a variety of arboreal activities including vertical climbing and suspension. In a comparison with mountain gorillas, chimpanzees continued these activities at higher rates and over a longer period of development [42]. In a varied locomotor repertoire, the direction and magnitude of the biomechanical forces acting on the knee will vary in accordance with the position and rotation of the knee joint [31]. Lovejoy [40] suggested that the shape of at least the lateral condyle in chimpanzees reflects the fact that there are equivalent joint stresses throughout the full range of flexion and extension of the knee joint; this is in opposition to humans, where joint stress is predictably highest during the last 20 degrees of extension [43]. The maintenance of a varied locomotor profile throughout ontogeny might then reasonably result in the retention of a particular distal femoral shape throughout ontogeny that is adapted for resisting biomechanical forces of variable direction and magnitude.

While there are differences among the adult taxa, the adult forms of all three taxa are more similar in shape to one another than their juvenile forms. One possibility is that these taxa become more similar over ontogeny as the result of epigenetic factors relating to terrestrial substrate use. In a study of the ontogeny of the tibiotalar joint in a variety of catarrhines, Turley and Frost [44] found shape convergence in the adult age classes of some phylogenetically distinct primate lineages that had similar substrate use. Similarly, these authors found divergence in phylogenetically close lineages (e.g., *Pan paniscus* and *Pan troglodytes*) in the adult age classes that use different substrates. They concluded that these similarities and differences represent evidence of epigenetic factors related to substrate use that influence joint shape congruence.

Finally, it should be noted that if the distal femur has multiple developmental modules (e.g., the medial and lateral condyles or the patellar articular surface and the condyles), then each module could be affected differentially and changes in a portion of the distal femur could be due to heterochrony. The analyses presented here address the complete shape of the distal femur and would be insufficient to uncover heterochrony in a single module, if modules do exist. Similarly, if there were a change in developmental timing that occurred before the youngest individuals in this analysis it would be difficult to detect with these analyses. These may be fruitful avenues for future research.

### Evidence for peramorphosis in the hominin distal femur

While more data need to be collected on a larger ontogenetic sample of modern humans (particularly individuals between 7 and 12 years of age), the overall results of these analyses indicate that the morphological pattern of some of the fossil individuals cannot be easily explained by changes in developmental rates in modern humans. The evidence presented here does not preclude the possibility that the morphological transition from early *Homo* to modern *Homo sapiens* is a result of heterochrony (Table 5 and Fig 4); however, these data do indicate that the morphological differences between *Homo* and particularly *A*. *afarensis* are not easily accounted for by heterochrony.

If the morphology of *Australopithecus* simply represented an early cessation of development as compared to *Homo sapiens*, then A.L. 333–4, A.L. 129-1a, and Sts 34 should have been most similar to the younger *Homo sapiens* specimens; instead, both A.L. 333–4 and A.L. 129-1a were more similar to *Pan* and *Gorilla* than to modern humans (Fig 4 and Table 5). In addition, KNM-ER 3951, a hominin of indeterminate genus and species, was also more similar to the *Pan/Gorilla* developmental trajectories than *Homo* (Fig 5). If the morphological variation in Plio-Pleistocene hominin distal femora is not due to differences in developmental patterning, then perhaps there was some change in the underlying regulatory genes that pattern the shape of the distal femur in bipedal hominins versus quadrupedal apes. While the forelimb and hindlimb are serially homologous structures governed by common *Hox* genes [45], [46], [47], [48], [49], there are genes that specifically affect hindlimb morphogenesis, specifically *Tbx4*, *Pitx1*, and *Pitx2* [50], [51], [52], [53], [54], [55]. It has been hypothesized that *Pitx1* and *Pitx2* function in hindlimb outgrowth, whereas *Tbx4* functions more for hindlimb shape [55], [56]. Mutations in the *Tbx4* genes have led to developmental abnormalities in the musculoskeletal system of the hindlimb [57], [58], [59], [60], [61], [62]. Additionally, GDF5 is a bone morphogenic protein that functions in the development of joints throughout the skeleton [63], [64], [65], [66]. Recently, separate GDF5 enhancers have been shown to affect very specific growth plates and individual diarthroses in the postcranial skeleton [67]. Changes in any of these genes could affect the patterning of the morphology of the distal femur and could be further modified epigenetically, as suggested by Lovejoy et al. [8].

The shape of all other fossil distal femora could be accommodated by the modern human ontogenetic trajectory. WT 15000—the only adolescent fossil individual—is most similar to adolescent modern humans whereas KNM-ER 1592, KNM-ER 1472, KNM-ER 1481, Sts 34, and Omo 1 –all adult specimens–are most similar to adult *Homo sapiens* (Fig 4 and Table 5). Thus, with regard to most of the fossils attributed to the genus *Homo* (as well as *A*. *africanus*, as exemplified by Sts 34) adult fossils appear most like adult *Homo sapiens* and the adolescent fossil appears most like adolescent *Homo sapiens*. This indicates that at least some fossil hominins have a similar pattern of development as modern *Homo sapiens* for this component of the knee joint. But, if rates of dental development are used as a proxy for maturation rates, it would indicate that these fossil hominins reach this same adult shape over a much shorter period of time than anatomically modern humans, as *A*. *africanus* is expected to reach adulthood at approximately 12 years, *Homo habilis* at approximately 15.5 years, and early *Homo erectus/ergaster* at 17 years [68].

## Conclusions

*Homo*, *Gorilla*, and *Pan* do not follow a similar developmental trajectory (Figs 2 and 5). Both *Homo* and *Gorilla* significantly change in shape as they grow, but in different manners, whereas *Pan* individuals do not greatly change shape in the distal femur as they age (Figs 3 and 4; Table 4). The earliest hominins in the sample, *Australopithecus afarensis* (represented by A.L. 129-1a and A.L. 333–4) cannot be accommodated by the human developmental trajectory. Their shape is better matched to *Gorilla* (Table 5) or the *Gorilla/Pan* ontogenetic trajectory (Fig 5). Thus, the hypothesis that the transition from an early hominin, *A*. *afarensis-*like, distal femoral shape to a modern human distal femoral can be explained by a heterochronic shift is rejected. Most other fossil hominins can be accommodated by the *Homo* ontogenetic trajectory and are most similar to specimens of the appropriate age group (in other words, adults look like adults and adolescents look like adolescents [Table 5 and Fig 5]), contra Tardieu [12], [13]. However, this does lend support for the importance of heterochronic shifts in human evolution as development would need to be either be slowed (neotony) or start much earlier (pre-displacement) in order for humans to achieve the same shape as earlier fossil hominins over a much longer period of time. However, the hypothesis that the modern human distal femur is the result of peramorphosis is also rejected.
